# Screening breast MRI in high-risk women undergoing prophylactic mastectomy

**DOI:** 10.1186/bcr3316

**Published:** 2012-11-09

**Authors:** SE McWilliams

**Affiliations:** 1 Guy's and St Thomas' NHS Foundation Trust, London, UK

## Introduction

High-risk women with genetic predisposition for breast cancer are increasingly being offered bilateral prophylactic mastectomy for risk reduction. If an incidental tumour is found at surgery, the patient cannot then undergo sentinel node biopsy of the axilla and has to have a full axillary clearance associated with morbidity of lymphoedema often in young women. We look at the use of screening MRI in 16 women at our institution prior to surgery to see how this affected management.

## Methods

Sixteen women aged 25 to 33 years underwent breast MRI prior to prophylactic mastectomy. The women were high risk with proven BRCA 1 or 2 mutations. The images were independently double-reported by two certified board breast radiologists. The patients also underwent bilateral mammogram.

## Results

Of the 16 women, one patient had a small tumour selected on MRI that was occult on conventional imaging and was able to undergo sentinel node biopsy. In the remaining 15 patients, one small 3 mm lesion was found at surgery and the patient had to have axillary dissection.

## Conclusion

Prophylactic mastectomy precludes the use of sentinel node biopsy as the breast is required for this procedure in order for the dye and tracer to be injected into the breast. Screening MRI prior to prophylactic mastectomy is essential in order for a sentinel node to be feasible in case a small tumour is present occult on conventional imaging.

